# Potential biomarkers and immune cell infiltration involved in aortic valve calcification identified through integrated bioinformatics analysis

**DOI:** 10.3389/fphys.2022.944551

**Published:** 2022-12-15

**Authors:** Xiaoshuo Lv, Xiaohui Wang, Jingwen Liu, Feng Wang, Mingsheng Sun, Xueqiang Fan, Zhidong Ye, Peng Liu, Jianyan Wen

**Affiliations:** ^1^ Department of Cardiovascular Surgery, China-Japan Friendship Hospital, Beijing, China; ^2^ Graduate School of Peking Union Medical College, Beijing, China; ^3^ Peking University China-Japan Friendship School of Clinical Medicine, Beijing, China

**Keywords:** calcific aortic valve disease, immune cell infiltration, bioinformatics, machine learning, LASSO, SVM-RFE

## Abstract

**Background:** Calcific aortic valve disease (CAVD) is the most common valvular heart disease in the aging population, resulting in a significant health and economic burden worldwide, but its underlying diagnostic biomarkers and pathophysiological mechanisms are not fully understood.

**Methods:** Three publicly available gene expression profiles (GSE12644, GSE51472, and GSE77287) from human Calcific aortic valve disease (CAVD) and normal aortic valve samples were downloaded from the Gene Expression Omnibus database for combined analysis. R software was used to identify differentially expressed genes (DEGs) and conduct functional investigations. Two machine learning algorithms, least absolute shrinkage and selection operator (LASSO) and support vector machine-recursive feature elimination (SVM-RFE), were applied to identify key feature genes as potential biomarkers for Calcific aortic valve disease (CAVD). Receiver operating characteristic (ROC) curves were used to evaluate the discriminatory ability of key genes. The CIBERSORT deconvolution algorithm was used to determine differential immune cell infiltration and the relationship between key genes and immune cell types. Finally, the Expression level and diagnostic ability of the identified biomarkers were further validated in an external dataset (GSE83453), a single-cell sequencing dataset (SRP222100), and immunohistochemical staining of human clinical tissue samples, respectively.

**Results:** In total, 34 identified DEGs included 21 upregulated and 13 downregulated genes. DEGs were mainly involved in immune-related pathways such as leukocyte migration, granulocyte chemotaxis, cytokine activity, and IL-17 signaling. The machine learning algorithm identified SCG2 and CCL19 as key feature genes [area under the ROC curve (AUC) = 0.940 and 0.913, respectively; validation AUC = 0.917 and 0.903, respectively]. CIBERSORT analysis indicated that the proportion of immune cells in Calcific aortic valve disease (CAVD) was different from that in normal aortic valve tissues, specifically M2 and M0 macrophages. Key genes SCG2 and CCL19 were significantly positively correlated with M0 macrophages. Single-cell sequencing analysis and immunohistochemical staining of human aortic valve tissue samples showed that SCG2 and CCL19 were increased in Calcific aortic valve disease (CAVD) valves.

**Conclusion:** SCG2 and CCL19 are potential novel biomarkers of Calcific aortic valve disease (CAVD) and may play important roles in the biological process of Calcific aortic valve disease (CAVD). Our findings advance understanding of the underlying mechanisms of Calcific aortic valve disease (CAVD) pathogenesis and provide valuable information for future research into novel diagnostic and immunotherapeutic targets for Calcific aortic valve disease (CAVD).

## Introduction

Calcific aortic valve disease (CAVD) is the most common cause of aortic valve stenosis worldwide and can cause clinical symptoms and left ventricular insufficiency due to severe obstruction of cardiac outflow (Lindman et al., 2016). The incidence of CAVD increases with age and is more than 1,000 per 100,000 at age >75 years. Furthermore, the prevalence of CAVD approximately 2.5 times over the past 30 years, resulting in a significant health and economic burden worldwide (Roth et al., 2020).

The complex pathogenesis of CAVD appears to encompass an active cellular process occurring in the aortic valve leaflets. This process produces progressive thickening of the leaflets and fibrous calcification remodeling, which is similar to atherosclerosis rather than being a simple degenerative change (Small et al., 2017; Xu et al., 2020). In addition, this pathogenesis involves initiation and propagation phases, which are dominated by different mechanisms (Schlotter et al., 2018). Lipid infiltration and inflammation play an important role in the first phase, while the transformation of aortic valve interstitial cells to osteoblast phenotypes is the key step in valve calcification in the propagation phase (Peeters et al., 2018; Pawade et al., 2019). A spectrum of molecular pathways drives the pathophysiological processes of calcification, including those involving lipoprotein a), angiotensin II (ANGII), Runt-related transcription factor 2 (RUNX2), Notch, and bone morphogenetic proteins (Peeters et al., 2018). Initial studies mainly focused on the role of these pathways in interstitial cells in CAVD, although recent studies have begun to investigate the role of inflammatory mechanisms in CAVD (Cho et al., 2018). For instance, the splicing product of signal transducer and activator of transcription 3 (STAT3), STAT3β, can antagonize calcification induced by macrophages (Raddatz et al., 2020). In addition, hydrogen sulfide can inhibit nuclear factor-κB (NF-κB) signaling, which is a critical inflammation mediator of the Runx2 pathway in calcification (Éva Sikura et al., 2021).

Although existing *in vivo* and *in vitro* studies advanced our understanding of CAVD, the underlying pathophysiological mechanisms are not fully understood. In addition, there is still no pharmacological therapy that effectively reduces CAVD onset or delays the progression of CAVD. For most symptomatic CAVD patients, the only treatment is surgery or transcatheter aortic valve replacement (de Oliveira Sá et al., 2020). Therefore, it is essential to explore the molecular mechanisms involved in CAVD pathogenesis to find new potential therapeutic targets.

Recent advances in gene chip technology helped identify new and important biomarker genes related to disease mechanisms that might act as diagnostic and treatment targets. To this end, we downloaded microarray datasets related to CAVD from the Gene Expression Omnibus (GEO) database. We analyzed differentially expressed genes (DEGs) and pathways between CAVD and normal aortic valve samples using bioinformatics methods. The key feature genes of CAVD were screened and identified by machine learning algorithms and confirmed by external datasets and human aortic tissue samples. In addition, we used CIBERSORT software to speculate the types and proportions of various immune cells in CAVD valve samples. The findings provide valuable information for future research on novel diagnostic and immunotherapeutic targets for CAVD.

## Materials and methods

### Microarray data

Before GEO database retrieval, the inclusion criteria of sequencing datasets were formulated as follows: 1. The experimental type was mRNA microarray sequencing (to facilitate subsequent data merging); 2. Sequencing samples included CAVD and normal aortic valve samples, and both CAVD and control samples were tricuspid aortic valves; 3. Complete mRNA expression matrix files can be obtained. According to this dataset inclusion criteria, matrix files of GSE12644, GSE51472, GSE77287, and GSE83453 datasets were downloaded from the GEO database. GSE12644 and GSE51472 datasets were both based on the GPL570 platform (Affymetrix Human Genome U133 Plus 2.0 Array), while the GSE77287 and GSE83453 datasets were based on GPL16686 (Affymetrix Human Gene 2.0 ST Array) and GPL10558 (Illumina HumanHT-12 V4.0 expression bead chip), respectively.

To exclude the influence of the bicuspid aortic valve on the results of this study, the samples with bicuspid valves were excluded during sample inclusion. All calcified aortic valves were taken from patients undergoing aortic valve replacement surgery. The normal valves in the control group were taken from heart transplant patients or patients with ascending aortic disease (aortic dissection or aortic aneurysm) requiring aortic valve replacement. Three microarray datasets from different countries (including the GSE12644, GSE51472, and GSE77287 datasets) were merged into a metadata cohort for further integration analysis. The GSE83453 dataset was used as a validation cohort.

### Batch correction and DEG screening

The “sva” R package was used to filter out any batch effects resulting from combination of the three datasets. Expression values were normalized using the “limma” package in R software (version 4.1.2; https://www.r-project.org/) to ensure expression levels had a similar distribution among a set of arrays. The “limma” package also was used to screen for DEGs, and samples with an adjusted false discovery rate (FDR) *p* < 0.05 and |log2 fold change (FC)| > 1 were considered DEGs.

### Functional enrichment analysis

Gene Ontology (GO) and Kyoto Encyclopedia of Genes and Genomes (KEGG) pathway enrichment analyses of DEGs were further explored with the “ClusterProfiler” and “org.Hs.eg.db” packages, and the adjusted *p*-value cutoffs were set to 0.05. To illustrate the relationships between enriched terms, Metascape (https://metascape.org/) was used to annotate and integrate the GO and KEGG analysis results. Additionally, gene set enrichment analysis (GSEA) was used to identify the most significant functional terms in CAVD samples and high SCG2 and CCL19 expressed samples after regrouping. The reference gene sets used for GSEA were “H.all.v7.5. Symbols.gmt”. The cutoff for GSEA was set as adjusted *p* < 0.05.

### Key feature genes screening

Two machine learning algorithms, least absolute shrinkage and selection operator (LASSO) logistic regression and support vector machine-recursive feature elimination (SVM-RFE), were used to perform feature selection to screen crucial genes with diagnostic utility. LASSO is a regression analysis algorithm that uses regularization to improve accuracy and was carried out using the “glmnet” package in R. SVM-RFE refers to a supervised machine learning method by complying with a support vector machine, which was adopted for finding optimal variables through the deletion of SVM-generated eigenvectors through the “e1071” package in R. Both machine learning techniques are widely used for classification and regression. Overlapping genes obtained from the two classification model algorithms were considered feature genes as potential biomarkers and were further analyzed.

### Diagnostic value and related biological process of identified key genes

For in-depth tests of the diagnosis efficacy of key genes, we generated receiver operating characteristic (ROC) curves using the metadata cohort of aortic valve samples. The area under the ROC curve (AUC) was used to determine the diagnostic effectiveness in discriminating CAVD from normal samples. Moreover, we verified the expression of key genes in the validation cohort (GSE83453) and evaluated the accuracy of key genes using ROC curves.

To further reveal the biological processes behind the identified key genes, we separately regrouped the samples according to the expression levels of the key genes. We divided the samples with gene expression levels higher than the median value into high expression groups, and the samples with gene expression levels lower than the median value into low expression groups, and then performed GSEA functional enrichment analysis on the high expression groups respectively using the reference gene sets of “c2. cp.kegg.v7.5.1. symbols.gmt” and “c5. go.bp.v7.5.1. symbols.gmt”.

### CIBERSORT analysis of immune cell infiltration

The CIBERSORT (https://cibersortx.stanford.edu/) algorithm (Newman et al., 2015) was used to evaluate differential immune cell infiltration between normal and CAVD aortic valve samples. The “LM22” gene file provided by CIBERSORT was used to define and infer the relative proportions of 22 types of immune cells in the gene expression data. The default signature matrix of 1000 permutations was used in this algorithm. To ensure confidence in the results, only data with deconvolution *p* < 0.05 were retained. After data processing and filtering, 18 cases of normal control and 23 cases of CAVD data were included in the subsequent analysis. The Wilcoxon test was used to estimate differences in immune cell infiltration between the CAVD and the normal group.

Correlations between immune cell subtypes and correlations between key genes and predicted immune cell levels were calculated using the “corrplot” R package (Spearman’s rank correlation method). Correlation coefficients |r | > 0.7 were considered strong, 0.5–0.7 moderately strong, 0.3–0.5 weak to moderately strong, and <0.3 weak. *p* < 0.05 means that the correlation is statistically significant. The “ggplot2” and “ggpubr” R packages were used for result visualization.

### Validation analysis at the single-cell level

The public single-cell sequencing dataset SRP222100 (2 normal and 4 CAVD samples) raw FASTQ data was downloaded from the SRA database, and gene expression files were subsequently obtained using the Cell Ranger (v6.1.2) following the previous standard data processing pipeline. Downstream analysis was performed using Seurat (v4.0.6) in the R environment, filtering out cells expressing more than 5% mitochondrial genes and retaining only cells expressing 200–3000 genes. The filtered data were removed batch effects through the “Harmony” method and the top 2000 variable genes for all samples were used to combine the samples into one object.

Subsequently, the data was log-transformed and scaled to unit variance and zero mean. Principal component analysis (PCA) was performed with a resolution of 0.4 to identify clusters and to perform t-distributed stochastic neighbor embedding (tSNE). DEGs across different cell clusters were identified with the “FindAllMarkers” function of Seurat. According to the highly expressed classical cell marker genes of each subgroup, and combined with the automatic cell type annotation package “SingleR”, different clusters were finally annotated as known cell types. The tSNE plot and cell type heatmap were plotted using “Seurat” R package, and the expression of candidate genes was displayed by heatmap using the “ComplexHeatmap” R package. GSEA analysis was performed for each cell cluster using the “singleseqGset” R package.

### Human specimen collection and immunohistochemical staining

The study was approved by the Medical Ethics Committee of China-Japan friendship hospital in Beijing, China (2019-25-1), and informed consent of all patients was obtained. Seven calcified aortic valve tissue samples were obtained from CAVD patients undergoing aortic valve replacement, and six non-calcified aortic valve tissue samples were obtained from patients with ascending aortic disease (aortic dissection or aortic aneurysm) requiring aortic valve replacement. The Paraffin-embedded tissue samples were cut into continuous sections with a thickness of 4 μm. Subsequently, sections were subjected to hematoxylin-eosin (H&E) staining and immunohistochemical staining. Sections were deparaffinated, blocked, and incubated with the primary anti-SCG2 antibody (Proteintech, 20357-1-AP) or Anti-CCL19 Antibody (Proteintech, 13397-1-AP) at 4°C overnight. Image-Pro Plus 6.0 Software (IPP 6.0, Media Cybernetics, United States) was utilized to measure the total tissue area and integrated optical density (IOD) of the target genes, which were stained yellow-brown. The intensity of gene expression was presented as IOD per unit area and the independent *t*-test was performed by R to analyze the difference between the two groups. *p* < 0.05 was considered statistically significant.

### Statistical analysis

All bioinformatics analyses were performed in R version 4.1.2 and its corresponding packages, and *p*-value or adjusted *p*-value <0.05 was considered statistically significant. SPSS version 26 and R software were used to analyze clinical characteristics and quantitative data of immunohistochemical staining results. Normally distributed continuous variables were depicted as means ± standard deviation (SD) and were compared between groups with an unpaired student’s t-test. Categorical variables were described as frequency (percentages) and compared between groups using Fisher’s exact test. For all statistical analyses, *p* < 0.05 was considered statistically significant.

## Results

### Data preprocessing and identification of DEGs

**FIGURE 1:**
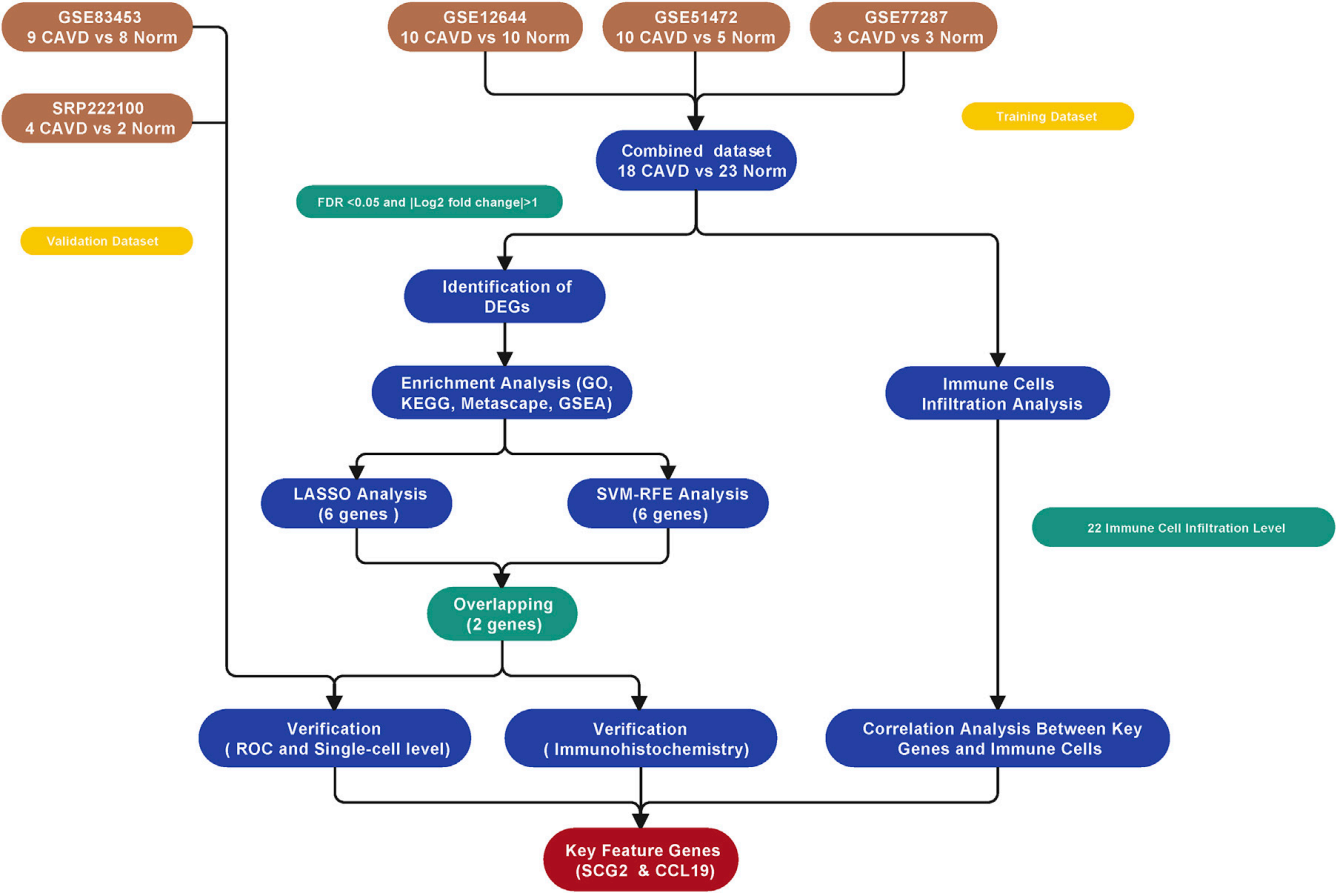
Flowchart for analysis in this study.

**FIGURE 2:**
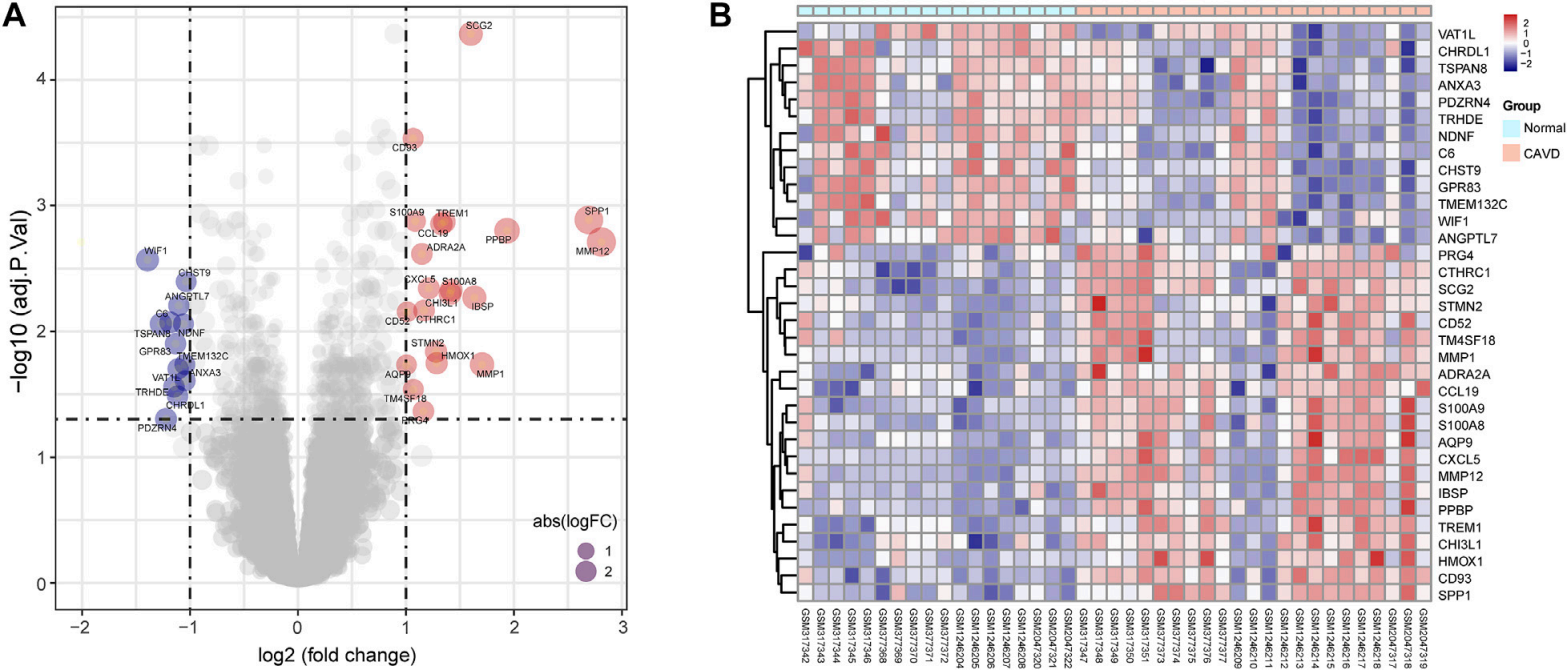
Differentially expressed genes (DEGs) between calcific aortic valve disease (CAVD) and normal aortic valve samples. **(A)** Volcano plot showing DEGs between CAVD and normal groups after combined analysis of GSE12644, GSE51472, and GSE77287 datasets with R software. The *x*-axis represents the fold change (log scaled), and the *y*-axis represents adjusted *p*-value (log-scaled). Red dots represent upregulated genes, blue dots represent downregulated genes, and size of dots represent the fold change of gene expression. **(B)** Heatmap showing DEGs between CAVD and normal groups. Upregulated genes are in red; downregulated genes are in blue. The samples clustered in light blue are normal aortic valve group, and the samples clustered in light red are CAVD group.

The study workflow was illustrated in Figure 1. A total of 18 normal and 23 CAVD samples from the metadata cohort (GSE12644, GSE51472, and GSE77287) were used as the training dataset, while GSE83453 (8 normal and 9 CAVD samples) and SRP222100 (2 normal and 4 CAVD samples) served as the validation datasets **(** Table 1 **)**. After batch effect removal and data correction, all expression values were normalized (Supplementary Figure S1). Finally, we obtained 34 DEGs in the CAVD group: 21 upregulated and 13 downregulated genes (Figures 2A,B), while a detailed summary was listed in Supplementary Table S1.

**TABLE 1:** Datasets used in this study.

Year	Country	Accession	Group		Platform	Classification
			CAVD	Normal		
2008	Canada	GSE12644	10	10	GPL570	Training sets
2013	Finland	GSE51472	10	5	GPL570	
2016	South Korea	GSE77287	3	3	GPL16686	
2016	Canada	GSE83453	9	8	GPL10558	Validation sets
2020	China	SRP222100	4	2	ILLUMINA	

CAVD, calcific aortic valve disease.

### Functional enrichment analysis

**FIGURE 3:**
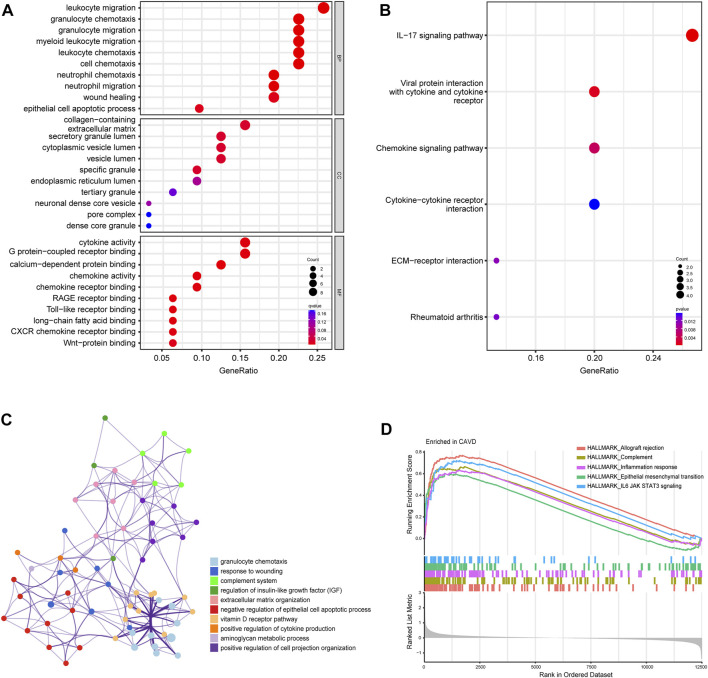
Biofunctional enrichment analysis of differentially expressed genes (DEGs). **(A)** Gene Ontology functional enrichment analyses of DEGs and the top 10 biological process (BP), cellular component (CC), and molecular function (MF) terms. **(B)** Kyoto Encyclopedia of Genes and Genomes pathway enrichment analyses of DEGs. The size of dots represents the count of DEGs enriched to the pathway, and the color from red to blue represents the change in *p*-value. **(C)** Network of the top 12 clusters of enriched terms in Metascape. Cluster identification is represented by color; similarity score is represented by edge thickness. **(D)** Results of gene set enrichment analysis (GSEA) showing the top five hallmark pathways most associated with CAVD pathology.

To further clarify the potential biological functions and processes of CAVD, we performed GO functional and KEGG pathway enrichment analyses of DEGs. GO terms were classified into three categories: biological process (BP), molecular function (MF), and cellular component (CC). The DEGs were mainly associated with the immune-related process such as leukocyte migration, granulocyte chemotaxis, and cytokine activity **(** Figure 3A **)**. Results of KEGG pathway analysis revealed enrichment in IL-17 signaling, viral protein interaction with cytokines, and chemokine signaling (Figure 3B). Metascape analysis showed the top 12 clusters with significantly enriched DEGs, including granulocyte chemotaxis and response to wounding (Figure 3C). GSEA analysis results showed that the top 5 items were allograft rejection, complement, inflammation response, epithelial mesenchymal transition, and IL6/JAK/STAT3 signaling (Figure 3D). Detailed results of all the functional enrichment analyses were listed in Supplementary Table S2. To further confirm the robustness of enriched biological processes, we carried out the same work in the validation set (GSE83453) and found that the top-ranked GO terms “collagens-containing extracellular matrix”, “Wnt-protein binding”; KEGG terms “Chemokine signaling pathway”, “Rheumatoid arthritis”; GSEA-Hallmark terms “allograft rejection”, “complement”, “inflammation response”, “epithelial mesenchymal transition” (Supplementary Figures S2A–C) were co-enriched biological processes in the metadata set and the validation set.

### Screening key feature genes

**FIGURE 4:**
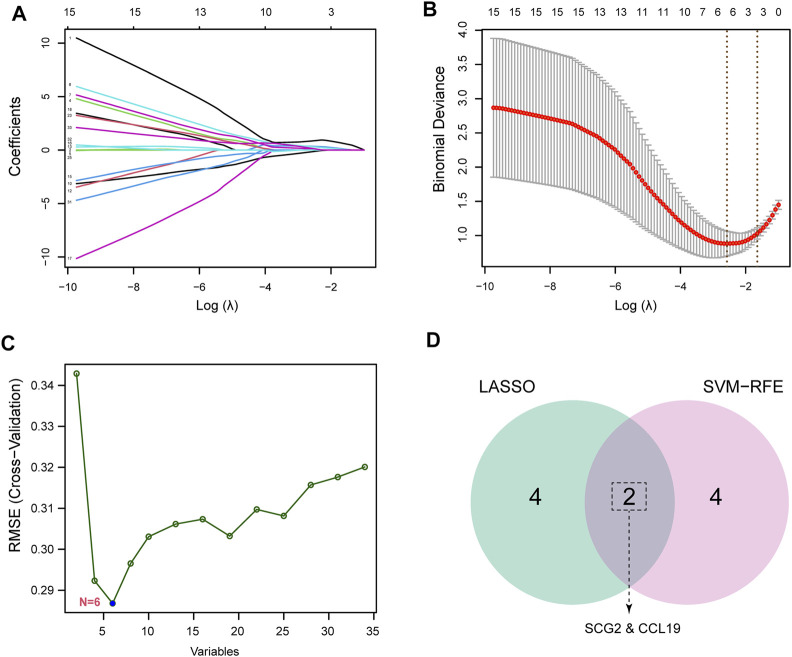
Screening for key feature genes by machine learning algorithms. **(A,B)** Feature genes identified using the least absolute shrinkage and selection operator (LASSO) logistic regression algorithm. Covariates are selected using the regularization parameter λ. **(C)** Support vector machine-recursive feature elimination (SVM-RFE) algorithm to screen feature genes. **(D)** Venn diagram demonstrating overlapping key feature genes screened by LASSO and SVM-RFE.

To identify potential biomarkers of CAVD, we applied two machine-learning algorithms to screen the feature genes. LASSO regression algorithm was used to narrow the DEGs, and 6 variables were identified as feature genes of CAVD (Figures 4A,B). Similarly, SVM-RFE algorithm also identified 6 feature genes from DEGs (Figure 4C). Two genes overlapped between the two algorithms, SCG2 and CCL19 (Figure 4D), which were ultimately identified as key feature CAVD genes.

### Verification of key feature genes and related functional analysis

**FIGURE 5:**
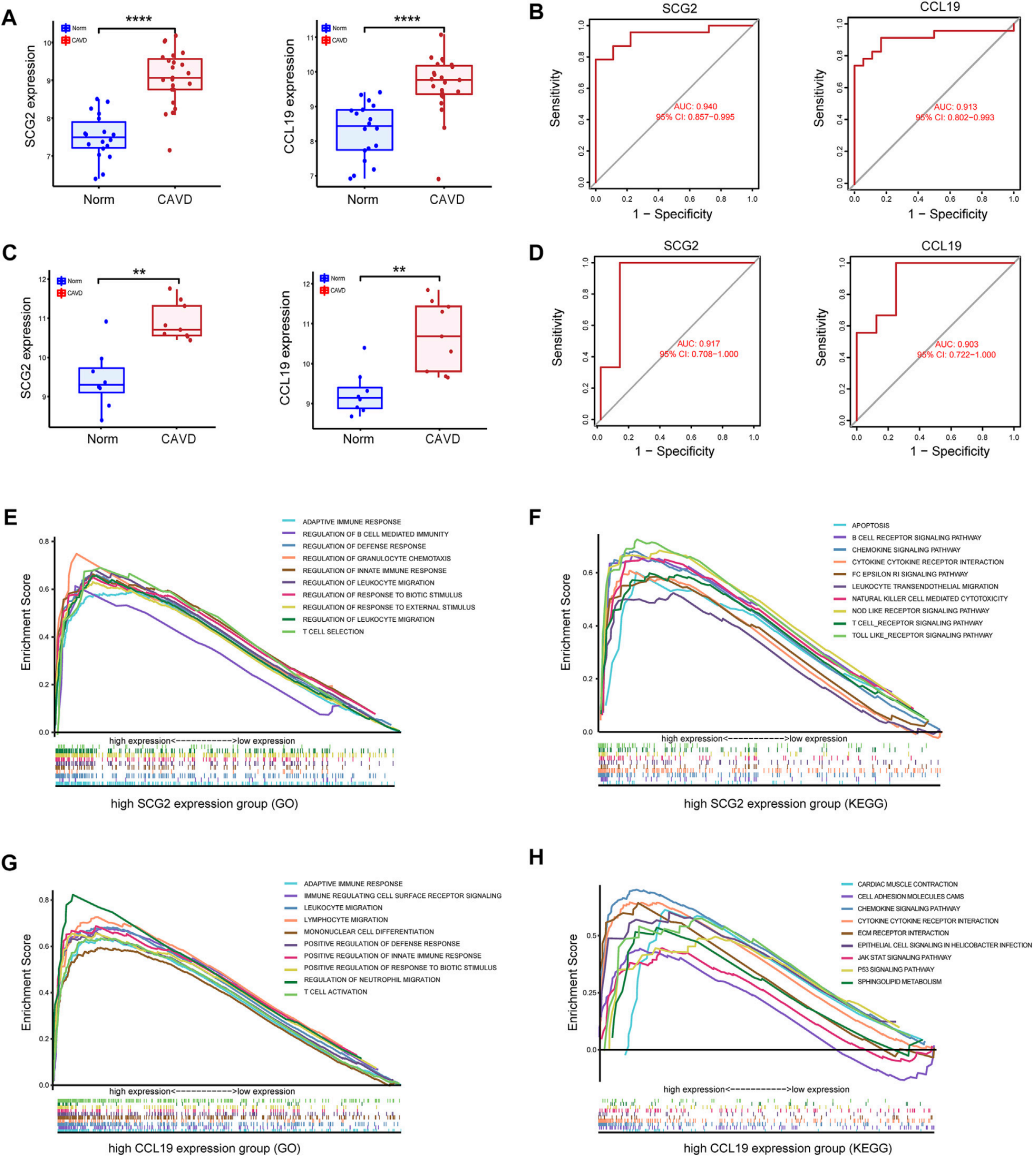
Validation of key genes SCG2 and CCL19. **(A,B)** Validation of the expression and receiver operating characteristic (ROC) curve of SCG2 and CCL19 in the merged metadata cohort. **(C,D)** Validation of the expression of SCG2 and CCL19 and ROC curve for evaluating the discriminatory accuracy of SCG2 and CCL19 in the validation dataset GSE83453. **(E,F)** GSEA analysis result show the top ten enrichment terms (GO and KEGG respectively) of regrouped high SCG2 expression group. **(G,H)** GSEA analysis result show the top ten enrichment terms (GO and KEGG respectively) of regrouped high CCL19 expression group. **p < 0.01, ****p < 0.0001.

Significantly high expression level of CCL19 of SCG2 in CAVD (Figure 5A) and ROC curves in the training datasets revealed their probability as valuable biological markers with AUCs of 0.940 and 0.913, respectively (Figure 5B), indicating a high diagnostic value. In addition, we validated the expression of the two genes and performed ROC analysis on the validation dataset. As expected, we detected significantly higher expression levels of SCG2 and CCL19 in CAVD samples than in the control group (*p* < 0.05) (Figure 5C). Moreover, the powerful discrimination ability of the two key genes was confirmed in the validation dataset, with an AUC of 0.917 for SCG2 and 0.903 for CCL19, indicating their high diagnostic ability (Figure 5D).

To gain insight into the possible biological pathways involved in SCG2 and CCL19, we performed GSEA analysis on samples regrouped according to the expression levels of SCG2 and CCL19. (Figures 5E–H) shows the top 10 biological processes (GO) and functional pathways (KEGG) after regrouping, most of which involve inflammatory pathways or immune regulation-related functions. Such as adaptive immune response, apoptosis involved in the high SCG2 expression group (Figures 5E,F); leukocyte migration, and chemokine signaling pathway involved in the high CCL19 expression group (Figures 5G,H).

### Immune cell infiltration

**FIGURE 6:**
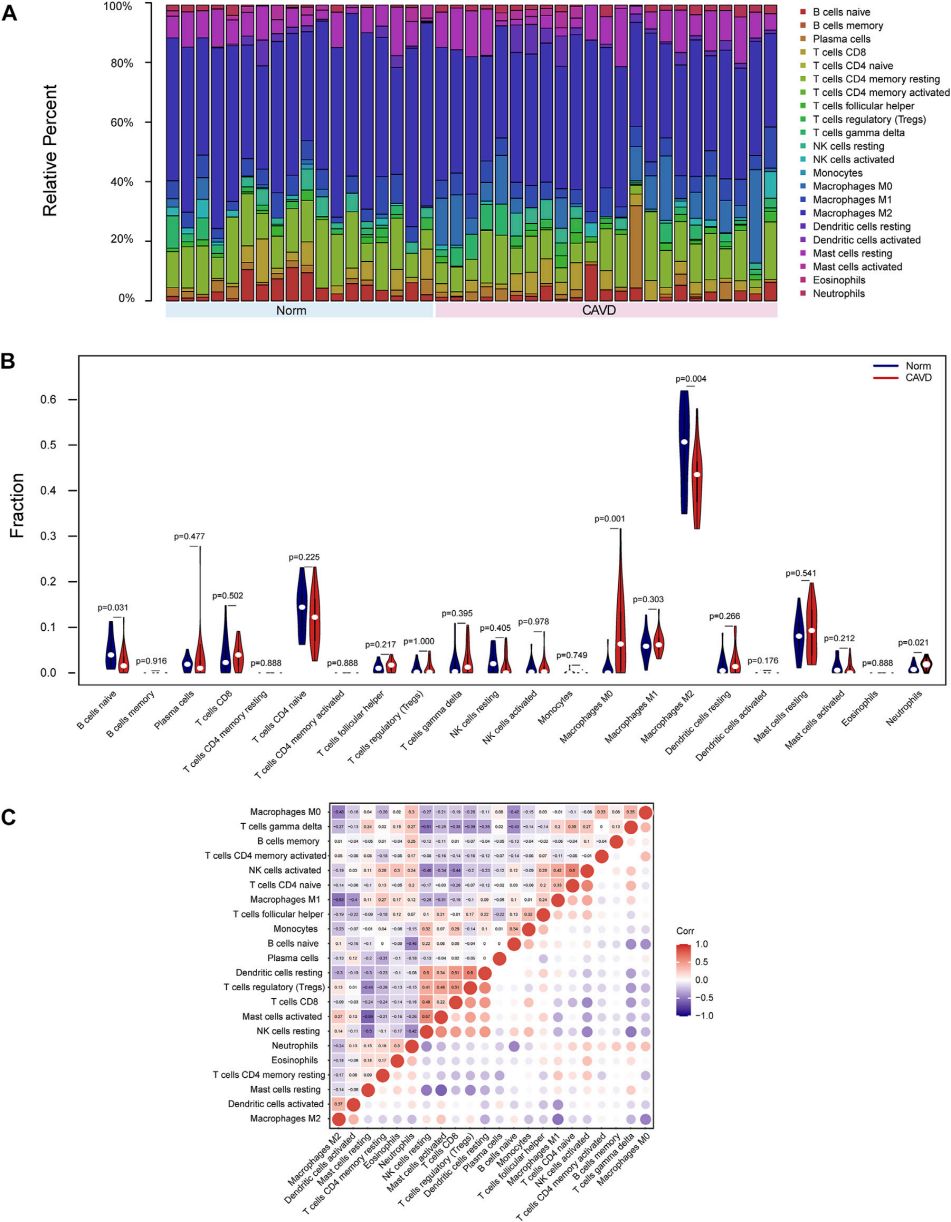
Landscape of immune cell infiltration of calcific aortic valve disease (CAVD). **(A)** Barplot of the proportions of 22 immune cell subpopulations in each group. The samples clustered in light blue are normal aortic valve group, and the samples clustered in light red are CAVD group. **(B)** Violin plot showing differentially infiltrated immune cells between normal (blue) and CAVD (red) groups. The vertical axis represents the relative proportion of immune cells. **(C)** Correlation matrix of 22 immune cell subtype compositions. Numbers in the small square represent Pearson’s correlation coefficient between the two immune cells in the horizontal and vertical coordinates. The color shades of the squares represent the degree of correlation.

Because functional enrichment analyses showed that the DEGs were mainly enriched in immune-related pathways, we used CIBERSORT to infer the pattern of immune cell signature in calcific aortic valves. Percentages of the 22 types of immune cells in each sample were shown in Figure 6A. The proportion of M2 macrophages, CD4 memory resting T cells, resting mast cells, M1 macrophages, and M0 macrophages ranked the top five in both groups of aortic valves.

The violin plot of immune cell signature differences demonstrated that CAVD patients had a significantly higher level of M0 macrophages and a lower level of M2 macrophages compared with the control group (Figure 6B). Furthermore, we performed correlation analysis of immune cells in aortic valves, with scores representing the degree of correlation. The correlation heatmap of immune cells revealed that resting dendritic cells were positively related to regulatory T cells (r = 0.6) and activated NK cells were positively related to CD4 naïve T cells (r = 0.6), whereas activated mast cells were negatively related to resting mast cells (r = −0.59), and M2 macrophages were negatively related to M1 macrophages (r = −0.53) (Figure 6C).

### Correlation analysis between key feature genes and immune cells

**FIGURE 7:**
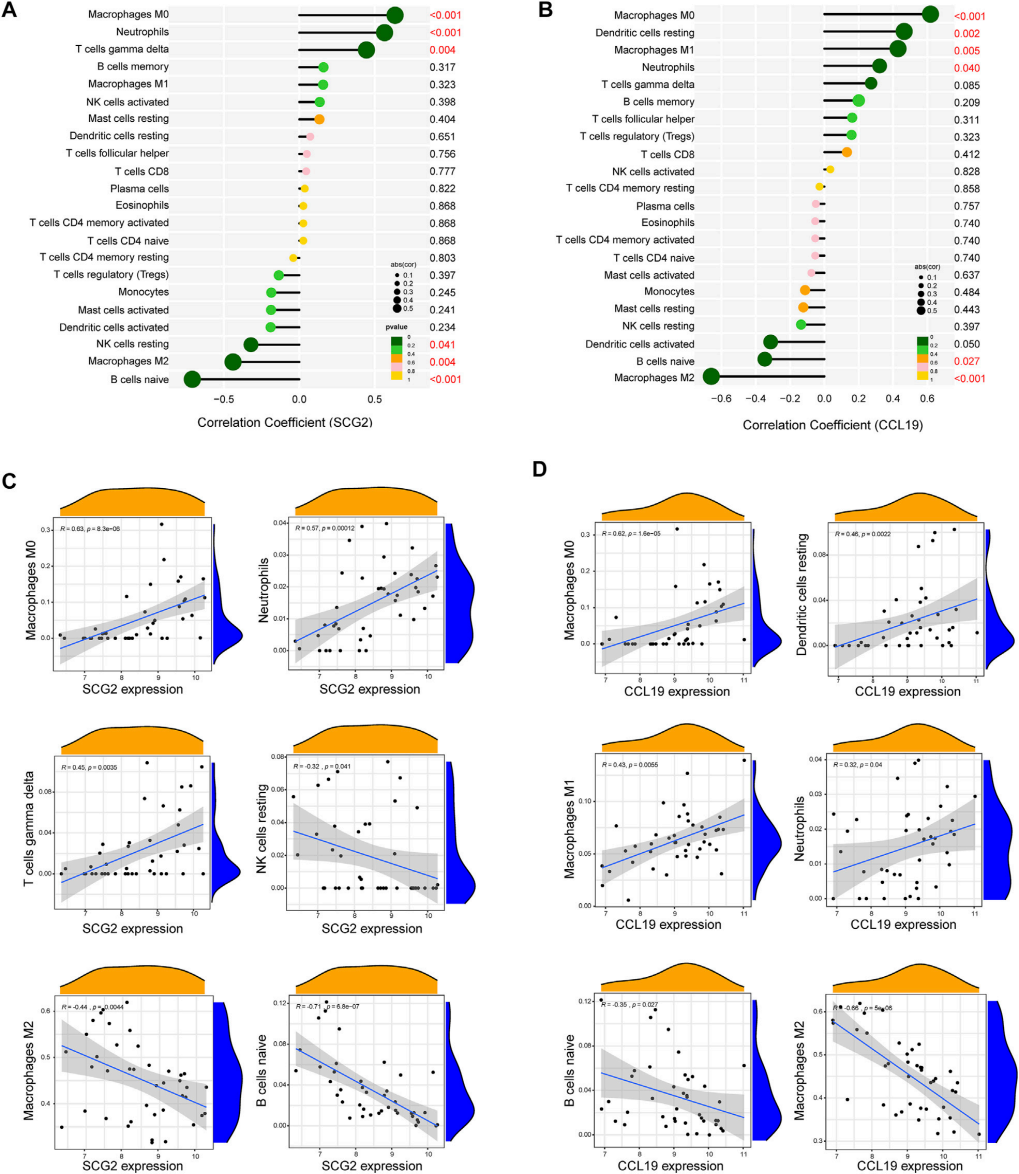
Correlation analysis between key genes and immune cells. **(A)** Correlation analysis for SCG2. **(B)** Correlation analysis for CCL19. Size of the dots represents the strength of the correlation between genes and immune cells, and color of the dots represents *p*-value. *p* < 0.05 was considered statistically significant. **(C,D)** Scatter plot showing the correlation between SCG2/CCL19 and immune cells. The *X*-axis represents the mRNA expression level of genes, the *Y*-axis represents the expression abundance of immune cells, and the R value represents the correlation coefficient.

As indicated by the correlation analysis, SCG2 was positively correlated with M0 macrophages (r = 0.635, *p* < 0.001), neutrophils (r = 0.565, *p* < 0.001), and gamma delta T cells (r = 0.445, *p* = 0.004) and negatively correlated with resting NK cells (r = −0.320, *p* = 0.041), M2 macrophages (r = −0.439, *p* = 0.004), and naïve B cells (r = −0.709, *p* < 0.001) (Figures 7A–C). CCL19 was positively correlated with M0 macrophages (r = 0.619, *p* < 0.001), resting dendritic cells (r = 0.465, *p* = 0.002), M1 macrophages (r = 0.429, *p* = 0.005), and neutrophils (r = 0.322, *p* = 0.040) and negatively correlated with naïve B cells (r = −0.347, *p* = 0.027), and M2 macrophages (r = −0.659, *p* < 0.001) (Figures 7B–D). All the above correlations were statistically significant, in which SCG2 was moderately to strongly correlated with M0 macrophages, neutrophils, and naive B cells; CCL19 was moderately to strongly correlated with M0 macrophages and M2 macrophages, and the rest correlations were weak to moderately correlated.

### Validation of key genes at the single cell level

**FIGURE 8:**
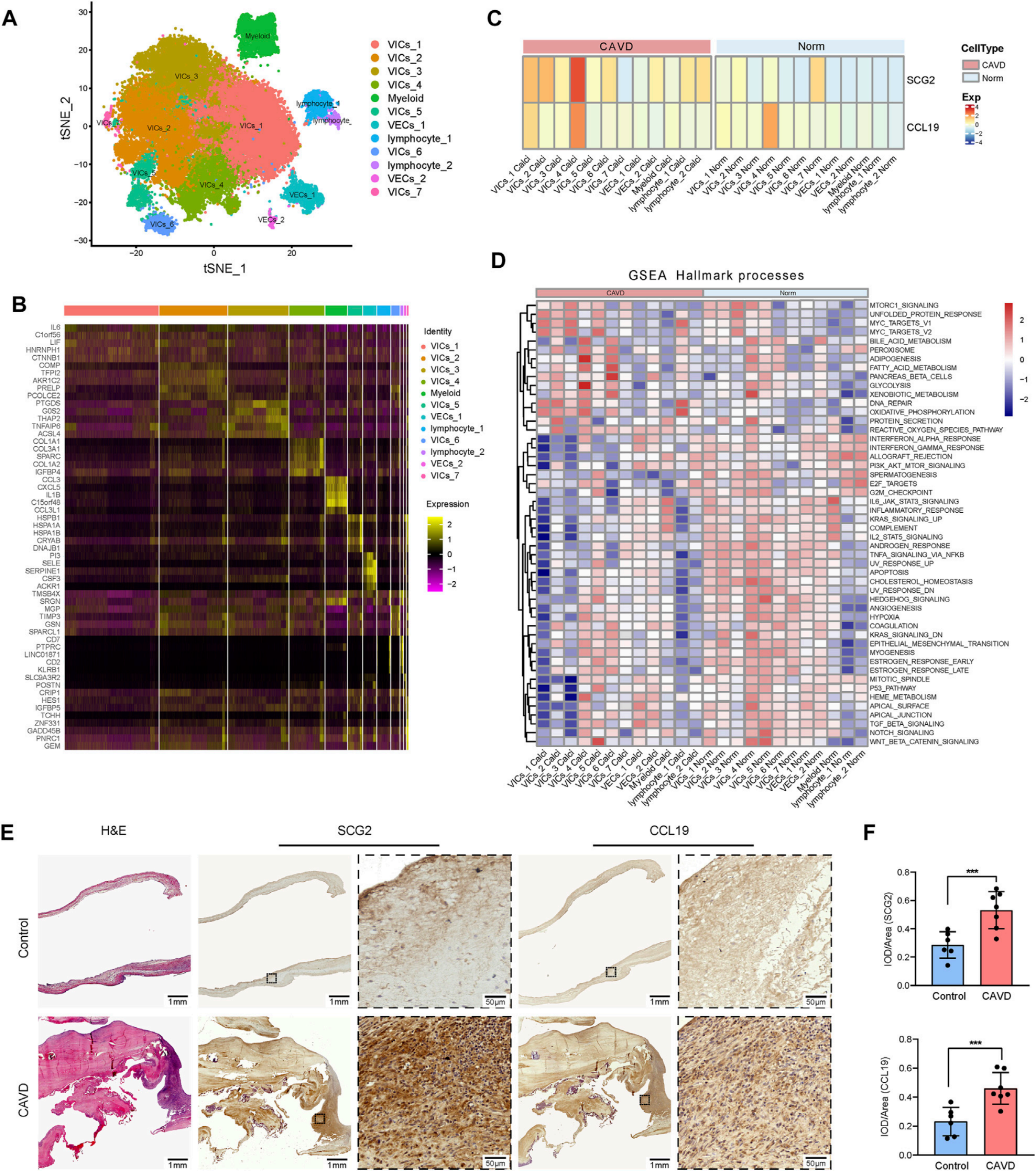
Validation at single cell level and tissue level. **(A)** tSNE visualization of clustering revealed 12 cell populations. Cell population identities were determined based on marker gene expression. **(B)** Heatmap of top 5 marker genes for each cell cluster. **(C)** Heatmap shows the expression of SCG2 and CCL19 in 12 clusters of cells in CAVD group and normal control group, respectively. The transition from blue to orange represents the increased expression level. **(D)** The heatmap of GSEA analysis results shows the hallmark functional pathways enriched in each of the calcified or normal aortic valve cell clusters. The transition from blue to red indicates the increased enrichment of pathways. **(E)** H and E staining and immunohistochemical staining of human aortic valve tissue sections. Macroscopic and micrographs views of immunohistochemical staining of SCG2 and CCL19 from left to right. **(F)** Histograms of quantitative immunohistochemical staining results. The expression of SCG2 and CCL19 was significantly increased in CAVD group. AUC: Area under the ROC curve; 95% CI: 95% confidence interval; Norm: normal; CAVD: calcific aortic valve disease; VICs: valvular interstitial cells; VECs: valvular endothelial cell; TCs: T cells. ***p < 0.001.

To confirm the role of SCG2 and CCL19 in CAVD and to clarify their distribution in various aortic valve cells, we analyzed single-cell sequencing data. The tSNE plot showed that 40,704 cells from 6 aortic valve samples (4 CAVD and 2 normal aortic valve samples) were grouped into 12 clusters, which were annotated as 7 clusters of valve interstitial cells (VICs), 2 clusters of valve endothelial cells (VECs), 1 cluster of myeloid cells, and 2 clusters of T cells (Figure 8A). Heatmap of the top marker genes per cluster was provided in Figure 8B. The results of gene heatmap showed that SCG2 and CCL19 were mainly expressed in VICs and lymphocytes, and the expression of SCG2 and CCL19 in the CAVD group was higher than that in the normal group. In particular, the expression of SCG2 and CCL19 were both significantly higher in the VICs 4 clusters of the CAVD group (Figure 8C).

Then we performed GSEA (Hallmark) analysis on each group of cells to further explore the functional pathways enriched in each group of cells. As shown in Figure 8D. MTORC 1 signaling, adipogenesis, glycolysis, WNT beta catenin signaling, etc. Were more active in VIC cells; DNA repair and oxidative phosphorylation were more active in lymphocytes. These results further reveal the biological processes that SCG2 and CCL19 may be involved in aortic valve calcification

### Demographic data of the patients and the expression of SCG2 and CCL19

Patients’ clinical characteristics including age, gender, body mass index (BMI), hypertension, diabetes mellitus, coronary heart disease (CHD), renal failure, statin use, and smoking were retrospectively collected and compared between groups. The results presented in Table 2 showed that all clinical characteristics were not statistically different between the two groups. Immunohistochemistry staining was used to assess the expression of SCG2 and CCL19 (Figure 8E), and omission of the primary antibody served as negative control (Supplementary Figure S3). Quantitative analysis by IPP software showed that the mean DOI of the tissue area (DOI/Area) of the two genes in CAVD samples was significantly higher than that in control samples (Figure 8F).

**TABLE 2:** Patient demographic data.

	Total	CAVD	Control	*p*-value
Patients	13	7	6	—
Male	10 (76.9%)	5 (71.4%)	5 (83.3%)	1
Age (years)	55.62 ± 2.014	55.86 ± 3.203	55.33 ± 2.591	0.903
BMI (kg/m^2^)	23.56 ± 0.559	23.133 ± 0.772	24.079 ± 0.833	0.423
Hypertension	5 (38.4%)	1 (14.2%)	4 (66.6%)	0.103
Yes				
Diabetes mellitus	1 (7.6%)	0 (0%)	1 (16.6%)	0.462
Yes				
Coronary heart disease	2 (15.3%)	2 (28.5%)	0 (0%)	0.462
Yes				
Chronic renal failure	2 (15.3%)	0 (0%)	2 (33.3%)	0.192
Yes				
Statin use	1 (7.6%)	1 (14.2%)	0 (0%)	1
Yes				
Smoker	5 (38.4%)	3 (42.8%)	2 (33.3%)	1
Yes				

Values are shown as mean ± SD, or n (%). BMI, body mass index.

## Discussion

CAVD is the third leading cause of cardiovascular disease, affecting about 25% of people >65 years of age (Virani et al., 2020; Wang and Pu, 2021). The only current treatment option for CAVD is cardiac valve implantation which has many defects such as high cost, short service life of the prosthesis, and the risk of prosthetic complications (Vahanian et al., 2012; Head et al., 2017). Therefore, it is of great practical significance to find the early diagnosis and intervention methods for CAVD. This study identified DEGs, enriched pathways, key genes, and immune cell infiltration patterns that provide a new comprehensive perspective to understand the pathological and immunological mechanisms of CAVD and offer clues to developing new therapeutic targets for CAVD.

For discovery, we obtained three gene expression profiles including 23 CAVD samples and 18 normal valve samples from the GEO database, and conducted an integrated analysis of the data. The 34 DEGs encompassed 21 upregulated genes and 13 downregulated genes. The results of functional enrichment analyses indicated that DEGs were mainly associated with inflammation and immune responses, such as leukocyte migration, granulocyte chemotaxis, and IL-17 signaling. Increasing numbers of studies have shown that inflammation exerts important roles in CAVD development and progression (Goody et al., 2020; Bartoli-Leonard et al., 2021). Consistent with recent work highlighting the complexity and interconnectivity of the immune system and valve cells, many biological processes associated with immune cell activation and migration and chemotactic signaling pathways were enriched in CAVD samples in this study. Similar to the pathological process of atherosclerosis (Sluiter et al., 2021), the initiation phase of CAVD involves chemotactic action mediated by cytokines such as TNFα, IL-1β, and IL-6 to activate and recruit immune cells to valve tissue (Isoda et al., 2010; Liu et al., 2017). Histopathologic studies have demonstrated that chronic inflammatory infiltration is closely related to valve tissue remodeling and neovascularization (Coté et al., 2013). This body of evidence suggests the importance of inflammation in the valvular calcification process.

In this study, SCG2 and CCL19 were identified as candidate feature genes by overlapping LASSO and SVM-RFE algorithms. Further ROC and expression verification confirmed their identity as CAVD feature genes with excellent diagnostic efficiency. To further verify the reliability of the results, we examined the expression of the two genes in aortic valves at the single-cell sequencing level and human specimens. Compared with non-calcified aortic valves, the expression of SCG2 and CCL19 was significantly increased in the CAVD samples. These results suggest that SCG2 and CCL19 are potential biomarkers of CAVD and may play an important role in the pathogenesis of CAVD.

SCG2 is a member of the chromogranin-secretogranin protein family of neuroendocrine secretory proteins (Troger et al., 2017). Previous studies have demonstrated that secretoneurin specifically activates a variety of cellular functions, including chemotactic migration of monocytes, eosinophils, fibroblasts, and smooth muscle cells, thereby modulating inflammatory responses (Wiedermann et al., 1999). Secretoneurin has been reported to be associated with many chronic inflammatory diseases, including chronic heart failure, essential hypertension, rheumatism, acute inflammatory syndrome, and neurogenic inflammation (Helle, 2010). However, the specific relationship between SCG2 and CAVD is still unclear. Only a few bioinformatic studies have reported the increased expression of SCG2 in CAVD samples (Sun et al., 2021), which is consistent with the results of this study, but there is still a lack of tissue level or experimental evidence. This study is the first to combine machine learning, single-cell sequencing data analysis, and histological examination of human specimens to provide more comprehensive evidence of the close association between SCG2 and CAVD. In previous studies, Fang C et al. reported that SCG2 impaired tumor growth and angiogenesis through degradation of HIF-1α (Fang et al., 2021); Luo MJ et al. showed that SCG2 can promote rapid wound healing under fasting conditions (Luo et al., 2020). These pieces of evidence indicated that SCG2 can regulate tissue remodeling, which may be important for the progression of CAVD. Functional enrichment analysis in this study showed that SCG2 was involved in a variety of biological functions in the process of valve calcification, such as granulocyte chemotaxis, leukocyte migration, regulation of epithelial cell apoptotic process, epithelial mesenchymal transformation (Supplementary Table S2), which provided direct clues to how SCG2 plays a biological role in CAVD.

CCL19 belongs to the chemokine superfamily, which plays a key role in controlling leukocyte recruitment during inflammatory responses (Yan et al., 2019). CCL19 and CCL21 are specific ligands of CCR7, which are expressed by various subsets of immune cells, and jointly regulate the induction of T cell activation, immune tolerance, and inflammatory responses (Rot and von Andrian, 2004; Yan et al., 2019). Several previous studies have reported that CCL19 and various proinflammatory cytokines are increased in calcified aortic valves and typically recruit CCR7+ dendritic cells and T lymphocytes to cause local inflammatory infiltrates (Raddatz et al., 2019; Broeders et al., 2022). In accordance with this, the immune cell infiltration analysis in this study suggested that CCL19 was positively correlated with M0/M1 macrophages, dendritic cells, and neutrophils; Functional enrichment analysis showed that CCL19 was involved in the chemokine signaling pathway and cytokine interaction. Previous evidence, together with the results of this study, demonstrated that CCL19-mediated inflammatory response plays an important role in CAVD. In addition to immune cells, the CCR7/CCL19 axis is expressed in airway smooth muscle cells, myofibroblasts, and fibroblasts (Rodríguez-Fernández and Criado-García, 2020), and is involved in biological processes such as tissue repair, endothelial-mesenchymal transition, and tumor metastasis (Xu et al., 2017; Chen et al., 2020). Wang et al. reported that CCR7 and CCL19 are expressed in the rheumatic mitral valve, and the CCR7/CCL19 axis may regulate the remodeling of rheumatic mitral valve injury by promoting the migration ability of VICs (Wang et al., 2015). Similarly, in the analysis of single-cell sequencing data in this study, VIC cells expressed higher levels of CCL19. These results suggest that in addition to proinflammatory function, CCL19 may play a variety of effects during tissue remodeling in the valve calcification process.

The results of this study showed that DEGs between CAVD and normal aortic valve samples were mainly enriched in immune and inflammatory-related pathways. To further investigate the effect of immune cells in the biological process of CAVD, we performed a comprehensive analysis of immune cell infiltration. The results predicted by CIBERSORT showed that M2 macrophages, CD4 memory resting T cells, resting mast cells, M1 macrophages, and M0 macrophages account for the main proportion of immune cells in aortic valves. This result is consistent with previous studies that macrophages, mast cells, CD4^+^ T cells, and CD8^+^ T cells constitute the main infiltrated immune cells in calcified aortic valves (Mathieu et al., 2015).

The important role of inflammation and immunity in CAVD has been widely discussed, as fibro-calcific remodeling and inflammation of the aortic valve are complex interrelated processes with important crosstalk (Lindman et al., 2016; Raddatz et al., 2019). Activated endothelial cells increase immune infiltration driven by the transendothelial migration of macrophages and T cells (Bartoli-Leonard et al., 2021). Macrophages are believed to be closely involved in the progression and severity of the cardiovascular disease. These cells can further differentiate into pro-inflammatory (M1) and anti-inflammatory (M2) phenotypes. M1 macrophages can produce iNOS, TNF-a, IL-6, IL-12, and MCP-1 and then promote the transmission of inflammatory responses, while M2 macrophages can produce anti-inflammatory cytokines such as TGF-β, IL-10, and CCL22 to play a protective role (Tabas and Bornfeldt, 2016; Fernandez et al., 2019). Previous studies have reported an increased expression of M1 markers and decreased expression of M2 markers in calcified valves compared with normal valves (Raddatz et al., 2019). Li G et al. demonstrated that the transition of macrophages to the M1 phenotype promotes aortic valve calcification (Li et al., 2017). In the present study, the results of CIBERSORT analysis showed a higher proportion of M0 macrophages and a lower proportion of M2 macrophages in the CAVD group compared with the normal group. It is noteworthy that the proportion of M1 macrophages also increased in CAVD samples, but it was not statistically significant. These results are generally consistent and together highlight the importance of macrophage and M1/M2 phenotypic switching in CAVD.

These results suggest that immune cells are closely related to the occurrence and progression of CAVD. However, the interactions between immune cells and the specific immune mechanism are still unclear and require further research. Interestingly, the results of this study showed that the key genes SCG2 and CCL19 were significantly positively correlated with M0 macrophages, while SCG2 and naïve B cells as well as CCL19 and M2 macrophages were significantly negatively correlated. These results provide new directions for further study of the immune mechanism of CAVD and the search for related therapeutic targets.

There are several limitations to this study. Firstly, some normal control samples in the included datasets and the clinical samples we used for validation were not taken from the aortic valves of healthy individuals, but from patients with ascending aortic disease requiring aortic valve replacement surgery. Diseases of the ascending aorta, such as aortic aneurysms and aortic dissection, may be associated with connective tissue disease, which may confound our results. Secondly, our sample size was still small after the combination of three microarray datasets, which may affect the accuracy of machine learning algorithm analysis. In future studies, we will continue to expand the sample size for validation, and further explore the mechanism of SCG2 and CCL19 in the occurrence and development of CAVD disease through *in vivo* and *in vitro* experiments.

## Conclusion

Integrated bioinformatic methods and machine learning algorithms identified key genes and pathways closely related to the biological process of CAVD. The expression of key genes SCG2 and CCL19 was validated using external datasets and human clinical tissue samples, confirming their elevated expression in the CAVD aortic valves. Furthermore, the CIBERSORT algorithm was used to speculate the types and proportions of various immune cells in aortic valve samples. Our findings advance our understanding of the pathogenesis of CAVD and provide valuable information for future research on new diagnostic and therapeutic targets of CAVD.

## Data Availability

The datasets presented in this study can be found in online repositories. The names of the repository/repositories and accession number(s) can be found in the article/Supplementary Material.
