# Supplementary material for: Potential biomarkers and immune cell infiltration involved in aortic valve calcification identified through integrated bioinformatics analysis
Source: Front Physiol. 2022 Dec 15;13:944551. doi: 10.3389/fphys.2022.944551 (PMC9797982; doi:10.3389/fphys.2022.944551)
Supplement: Supplementary file 1 [file DataSheet2.PDF]

Table S2: The detailed results of Functional enrichment analyses

GO functional enrichment analysis results

ONTOLOGY	ID	Description	pvalue	geneID
BP	GO:0071621	granulocyte chemotaxis	8.67E-07	SCG2/S100A9/TREM1/CCL19/PPBP/CXCL5/S100A8
BP	GO:0097530	granulocyte migration	1.42E-06	SCG2/S100A9/TREM1/CCL19/PPBP/CXCL5/S100A8
BP	GO:0030593	neutrophil chemotaxis	3.96E-06	S100A9/TREM1/CCL19/PPBP/CXCL5/S100A8
BP	GO:1990266	neutrophil migration	8.22E-06	S100A9/TREM1/CCL19/PPBP/CXCL5/S100A8
BP	GO:0097529	myeloid leukocyte migration	8.78E-06	SCG2/S100A9/TREM1/CCL19/PPBP/CXCL5/S100A8
BP	GO:0030595	leukocyte chemotaxis	9.92E-06	SCG2/S100A9/TREM1/CCL19/PPBP/CXCL5/S100A8
BP	GO:0050900	leukocyte migration	1.21E-05	SCG2/S100A9/TREM1/CCL19/PPBP/CXCL5/S100A8/HMOX1
BP	GO:0060326	cell chemotaxis	5.62E-05	SCG2/S100A9/TREM1/CCL19/PPBP/CXCL5/S100A8
BP	GO:0042060	wound healing	0.00484354	MMP12/ADRA2A/S100A8/TSPAN8/NDNF/HMOX1
BP	GO:1904036	negative regulation of epithelial cell apoptotic process	0.005767429	SCG2/NDNF/HMOX1
BP	GO:0030198	extracellular matrix organization	0.007617006	MMP12/IBSP/ANGPTL7/NDNF/MMP1
BP	GO:0043062	extracellular structure organization	0.007617006	MMP12/IBSP/ANGPTL7/NDNF/MMP1
BP	GO:0045229	external encapsulating structure organization	0.007617006	MMP12/IBSP/ANGPTL7/NDNF/MMP1
BP	GO:0006959	humoral immune response	0.008601198	S100A9/TREM1/PPBP/CXCL5/C6
BP	GO:0070486	leukocyte aggregation	0.010294857	S100A9/S100A8
BP	GO:0042742	defense response to bacterium	0.011841957	S100A9/TREM1/PPBP/S100A8/ANXA3
BP	GO:0061844	antimicrobial humoral immune response mediated by antimicrobial peptide	0.011841957	S100A9/PPBP/CXCL5
BP	GO:0017014	protein nitrosylation	0.011841957	S100A9/S100A8
BP	GO:0018119	peptidyl-cysteine S-nitrosylation	0.011841957	S100A9/S100A8
BP	GO:0051238	sequestering of metal ion	0.011841957	S100A9/S100A8
BP	GO:0002544	chronic inflammatory response	0.014065959	S100A9/S100A8
BP	GO:0070098	chemokine-mediated signaling pathway	0.014065959	CCL19/PPBP/CXCL5
BP	GO:0002523	leukocyte migration involved in inflammatory response	0.014628376	S100A9/S100A8
BP	GO:1904035	regulation of epithelial cell apoptotic process	0.01583109	SCG2/NDNF/HMOX1
BP	GO:1990868	response to chemokine	0.01583109	CCL19/PPBP/CXCL5
BP	GO:1990869	cellular response to chemokine	0.01583109	CCL19/PPBP/CXCL5
BP	GO:0002444	myeloid leukocyte mediated immunity	0.01618136	TREM1/HMOX1/ANXA3
BP	GO:0032103	positive regulation of response to external stimulus	0.016919549	SCG2/S100A9/CCL19/MMP12/S100A8
BP	GO:1903306	negative regulation of regulated secretory pathway	0.01709458	ADRA2A/HMOX1
BP	GO:0046916	cellular transition metal ion homeostasis	0.022518191	S100A9/S100A8/HMOX1
BP	GO:0001819	positive regulation of cytokine production	0.022871708	CCL19/MMP12/ADRA2A/CHI3L1/HMOX1
BP	GO:1904019	epithelial cell apoptotic process	0.024287746	SCG2/NDNF/HMOX1
BP	GO:0019730	antimicrobial humoral response	0.024287746	S100A9/PPBP/CXCL5
BP	GO:0030204	chondroitin sulfate metabolic process	0.024889255	CHST9/NDNF
BP	GO:0006022	aminoglycan metabolic process	0.026897505	CHST9/CHI3L1/NDNF
BP	GO:0045920	negative regulation of exocytosis	0.028443975	ADRA2A/HMOX1
BP	GO:0002446	neutrophil mediated immunity	0.028601621	TREM1/ANXA3
BP	GO:2000352	negative regulation of endothelial cell apoptotic process	0.028601621	SCG2/NDNF
BP	GO:0055076	transition metal ion homeostasis	0.028788625	S100A9/S100A8/HMOX1
BP	GO:0050654	chondroitin sulfate proteoglycan metabolic process	0.028788625	CHST9/NDNF
BP	GO:0006882	cellular zinc ion homeostasis	0.033082121	S100A9/S100A8
BP	GO:0055069	zinc ion homeostasis	0.035757955	S100A9/S100A8
BP	GO:0030574	collagen catabolic process	0.038474033	MMP12/MMP1
BP	GO:0014002	astrocyte development	0.039393174	S100A9/S100A8
BP	GO:0032496	response to lipopolysaccharide	0.039517193	S100A9/PPBP/CXCL5/S100A8
BP	GO:0010976	positive regulation of neuron projection development	0.039870346	S100A9/NDNF/STMN2
BP	GO:1901342	regulation of vasculature development	0.039870346	CHI3L1/ANGPTL7/HMOX1/ANXA3
BP	GO:1903034	regulation of response to wounding	0.040266083	SPP1/ADRA2A/TSPAN8
BP	GO:0031346	positive regulation of cell projection organization	0.040266083	S100A9/CCL19/NDNF/STMN2
BP	GO:2001233	regulation of apoptotic signaling pathway	0.040684475	SCG2/S100A9/S100A8/HMOX1
BP	GO:0002237	response to molecule of bacterial origin	0.042328759	S100A9/PPBP/CXCL5/S100A8
BP	GO:0018198	peptidyl-cysteine modification	0.042328759	S100A9/S100A8
BP	GO:0050832	defense response to fungus	0.042328759	S100A9/S100A8
BP	GO:0045766	positive regulation of angiogenesis	0.044916167	CHI3L1/HMOX1/ANXA3
BP	GO:1904018	positive regulation of vasculature development	0.044916167	CHI3L1/HMOX1/ANXA3
BP	GO:0009100	glycoprotein metabolic process	0.049016458	CCL19/MMP12/CHST9/NDNF
CC	GO:0062023	collagen-containing extracellular matrix	0.01698975	S100A9/S100A8/ANGPTL7/CTHRC1/PRG4
CC	GO:0034774	secretory granule lumen	0.01698975	S100A9/PPBP/S100A8/CHI3L1
CC	GO:0060205	cytoplasmic vesicle lumen	0.01698975	S100A9/PPBP/S100A8/CHI3L1
CC	GO:0031983	vesicle lumen	0.01698975	S100A9/PPBP/S100A8/CHI3L1
CC	GO:0042581	specific granule	0.01698975	CD93/CHI3L1/ANXA3
MF	GO:0048306	calcium-dependent protein binding	0.000789757	S100A9/S100A8/STMN2/ANXA3
MF	GO:0005125	cytokine activity	0.00123425	SCG2/SPP1/CCL19/PPBP/CXCL5
MF	GO:0008009	chemokine activity	0.001381976	CCL19/PPBP/CXCL5
MF	GO:0050786	RAGE receptor binding	0.001442137	S100A9/S100A8
MF	GO:0001664	G protein-coupled receptor binding	0.001442137	CCL19/PPBP/ADRA2A/CXCL5/CTHRC1
MF	GO:0035325	Toll-like receptor binding	0.001582882	S100A9/S100A8
MF	GO:0042379	chemokine receptor binding	0.001633285	CCL19/PPBP/CXCL5
MF	GO:0036041	long-chain fatty acid binding	0.001633285	S100A9/S100A8
MF	GO:0045236	CXCR chemokine receptor binding	0.002430352	PPBP/CXCL5
MF	GO:0017147	Wnt-protein binding	0.006138226	WIF1/CTHRC1
MF	GO:0048018	receptor ligand activity	0.006336457	SCG2/SPP1/CCL19/PPBP/CXCL5
MF	GO:0030546	signaling receptor activator activity	0.006336457	SCG2/SPP1/CCL19/PPBP/CXCL5
MF	GO:0005178	integrin binding	0.007258383	SPP1/IBSP/TSPAN8
MF	GO:0005504	fatty acid binding	0.007258383	S100A9/S100A8
MF	GO:0015144	carbohydrate transmembrane transporter activity	0.007258383	PPBP/AQP9
MF	GO:0005201	extracellular matrix structural constituent	0.010136614	CHI3L1/CTHRC1/PRG4
MF	GO:0008237	metallopeptidase activity	0.012611407	MMP12/MMP1/TRHDE
MF	GO:0033293	monocarboxylic acid binding	0.019663276	S100A9/S100A8
MF	GO:0005126	cytokine receptor binding	0.028417246	CCL19/PPBP/CXCL5
MF	GO:0030246	carbohydrate binding	0.028417246	CD93/CHI3L1/PRG4
MF	GO:0004222	metalloendopeptidase activity	0.037579038	MMP12/MMP1
MF	GO:0004983	neuropeptide Y receptor activity	0.038881499	GPR83
MF	GO:0031996	thioesterase binding	0.040691928	ADRA2A
MF	GO:0015166	polyol transmembrane transporter activity	0.040691928	AQP9
MF	GO:0004859	phospholipase inhibitor activity	0.040691928	ANXA3
MF	GO:0015250	water channel activity	0.040691928	AQP9
MF	GO:1901338	catecholamine binding	0.040691928	ADRA2A
MF	GO:0043177	organic acid binding	0.040691928	S100A9/S100A8
MF	GO:0001846	opsonin binding	0.043926867	CD93
MF	GO:0015631	tubulin binding	0.043926867	S100A9/S100A8/STMN2
MF	GO:0005372	water transmembrane transporter activity	0.043926867	AQP9
MF	GO:0055102	lipase inhibitor activity	0.047768272	ANXA3
MF	GO:0031690	adrenergic receptor binding	0.047768272	ADRA2A
MF	GO:0001848	complement binding	0.047768272	CD93
MF	GO:0004252	serine-type endopeptidase activity	0.047768272	MMP12/MMP1
MF	GO:0031406	carboxylic acid binding	0.047768272	S100A9/S100A8
MF	GO:0005355	glucose transmembrane transporter activity	0.047768272	PPBP
MF	GO:0015149	hexose transmembrane transporter activity	0.047768272	PPBP
MF	GO:0030021	extracellular matrix structural constituent conferring compression resistance	0.047768272	PRG4

KEGG pathway analysis results

ID	Description	pvalue	geneID
hsa04657	IL-17 signaling pathway	2.09E-05	S100A9/CXCL5/S100A8/MMP1
hsa04061	Viral protein interaction with cytokine and cytokine receptor	0.000742439	CCL19/PPBP/CXCL5
hsa04062	Chemokine signaling pathway	0.004812652	CCL19/PPBP/CXCL5
hsa04512	ECM-receptor interaction	0.011143609	SPP1/IBSP
hsa05323	Rheumatoid arthritis	0.012387461	CXCL5/MMP1
hsa04060	Cytokine-cytokine receptor interaction	0.015646775	CCL19/PPBP/CXCL5

GSEA (hallmark gene sets) analysis results

Description	setSize	enrichmentScore	NES	qvalues	leading_edge	core_enrichment

HALLMARK_ALLOGRAFT_REJECTION	117	0.769183195	2.90945	1.12E-09	tags=62%, list=14%, signal=54%	CCL19/TLR2/CCR1/CD2/LY86/CCL11/GPR65/THY1/SRGN/CXCL13/CXCL9/CCR5/CCL5/CCL4/CTSS/HCLS1/LCK/IL18/ITK/TLR1/PRF1/LCP2/CD86/FGR/LYN/CRTAM/CD8A/TRAT1/IRF8/WAS/CD28/CSK/CCR2/CD1D/CCL7/CXCR3/STAT4/ELF4/SIT1/TIMP1/IL18RAP/IL7/CDKN2A/STAB1/SPI1/MAP4K1/C2/ZAP70/GALNT1/CD80/TNF/ETS1/PF4/CD4/CD96/TLR6/IFNGR1/CD247/CCL22/EIF5A/SOCS1/LIF/CD40LG/BCL10/ICOSLG/ICAM1/IRF7/IRF4/JAK2/IL4R/F2/CCL2/SOCS5
HALLMARK_COMPLEMENT	116	0.664890323	2.508559	1.12E-09	tags=51%, list=15%, signal=44%	MMP12/S100A9/GZMK/MMP13/RASGRP1/CTSC/APOC1/CCL5/CR1/CTSS/PLEK/DOCK10/LCK/S100A12/LCP2/DPP4/LYN/CTSB/KYNU/FN1/PFN1/FCN1/WAS/SH2B3/CTSD/CTSH/RHOG/OLR1/TIMP1/CTSL/PIM1/LAP3/GRB2/ADAM9/C2/MMP14/LTF/PDGFB/NOTCH4/DUSP6/CA2/MSRB1/ADRA2B/LGMN/MMP8/CD40LG/FDX1/ITIH1/IRF7/JAK2/CTSV/MT3/CFB/F2/CSRP1/MMP15/DOCK4/LRP1/IRF1
HALLMARK_INFLAMMATORY_RESPONSE	129	0.632185095	2.420514	1.12E-09	tags=50%, list=13%, signal=44%	AQP9/C5AR1/CXCL8/TLR2/IL7R/RASGRP1/CD14/CXCL10/CXCL9/CD48/CCL5/GPR183/FPR1/LAMP3/LCK/IL18/TLR1/LCP2/PTGER4/LYN/CD69/NOD2/RGS1/CCR7/MSR1/CCL7/C3AR1/PDPN/CSF3R/RHOG/CCRL2/OLR1/SPHK1/TIMP1/IL18RAP/STAB1/PTAFR/MMP14/FFAR2/ADORA2B/IL18R1/RGS16/APLNR/CMKLR1/ADM/BDKRB1/RNF144B/CXCL6/CCL20/CCL24/AHR/CCL22/IFITM1/SLAMF1/OSM/LIF/ICOSLG/ICAM1/NMI/OSMR/CD70/MXD1/IRF7/IL4R
HALLMARK_EPITHELIAL_MESENCHYMAL_TRANSITION	110	0.60084854	2.255957	8.94E-08	tags=38%, list=7%, signal=36%	SPP1/MMP1/SCG2/CTHRC1/TNC/VCAM1/CXCL8/GREM1/MFAP5/THBS2/CXCL12/LRRC15/IGFBP4/THY1/SDC1/ADAM12/COMP/CRLF1/FN1/LUM/TAGLN/HTRA1/TIMP1/CADM1/ANPEP/NID2/MXRA5/LOX/THBS1/VEGFA/MMP14/IGFBP3/LOXL2/OXTR/P3H1/GLIPR1/CALD1/GPX7/NNMT/CXCL6/CD44/TPM2
HALLMARK_IL6_JAK_STAT3_SIGNALING	50	0.721331555	2.366577	3.67E-07	tags=60%, list=13%, signal=53%	HMOX1/TLR2/CCR1/CD14/CXCL10/CXCL13/CXCL9/CCL7/CXCL3/CSF3R/PIM1/IL7/SOCS3/A2M/MAP3K8/GRB2/ACVRL1/MYD88/IL18R1/TNF/PF4/CD44/IFNGR1/BAK1/CD38/IL9R/SOCS1/IL17RA/OSMR/IL4R
HALLMARK_INTERFERON_GAMMA_RESPONSE	117	0.522909761	1.977916	5.00E-05	tags=43%, list=14%, signal=37%	VCAM1/CXCL10/CXCL9/CCL5/FPR1/SLAMF7/LCP2/CD86/VAMP8/CD69/SECTM1/IRF8/CCL7/STAT4/PIM1/ISG20/IL7/LAP3/SOCS3/MYD88/GPR18/UPP1/METTL7B/IL18BP/CMKLR1/PNP/IRF5/MX2/MT2A/TRIM14/RNF31/EPSTI1/ISG15/CD38/XCL1/SOCS1/PELI1/OASL/ICAM1/NMI/IFI44/IRF7/IRF4/JAK2/IL4R/RNF213/CFB/PML/AUTS2/CCL2
HALLMARK_KRAS_SIGNALING_UP	135	0.476044056	1.850193	0.000212454	tags=29%, list=7%, signal=27%	SPP1/PPBP/IL7R/PLVAP/LAPTM5/SLPI/TLR8/CXCL10/LY96/TMEM158/CTSS/LCP1/CXCR4/GALNT3/IRF8/TSPAN13/C3AR1/ADAM8/NRP1/ERO1A/DOCK2/EPB41L3/MAFB/CD37/PLEK2/MAP4K1/MMP11/ACE/IKZF1/TRIB1/HSD11B1/IGFBP3/RGS16/GLRX/ETS1/CMKLR1/DUSP6/CA2/TMEM176B
HALLMARK_ANGIOGENESIS	26	0.735442635	2.148115	0.000371394	tags=38%, list=7%, signal=36%	SPP1/STC1/LUM/NRP1/OLR1/TIMP1/VEGFA/JAG2/PF4/CXCL6
HALLMARK_IL2_STAT5_SIGNALING	137	0.460824421	1.793632	0.000480068	tags=33%, list=13%, signal=29%	SPP1/EOMES/GPR65/CXCL10/CD48/CD86/CST7/IRF8/CTSZ/NRP1/RHOH/ENPP1/PIM1/TLR7/MAP3K8/MYO1E/HOPX/CDCP1/ADAM19/ALCAM/IL18R1/RGS16/CISH/PNP/GSTO1/CD83/CA2/MAPKAPK2/ICOS/CD44/IFNGR1/IGF2R/PHTF2/SYNGR2/SPRY4/AHR/AHCY/SOCS1/CKAP4/PTRH2/LIF/MXD1/PLEC/IRF4/IL4R
HALLMARK_TNFA_SIGNALING_VIA_NFKB	133	0.466655051	1.80462	0.000678418	tags=36%, list=13%, signal=32%	TNC/BCL2A1/TLR2/IL7R/CXCL10/CCL5/GPR183/CCL4/PLEK/IL18/PTGER4/KYNU/CD69/CXCL3/MARCKS/CCRL2/OLR1/SPHK1/DRAM1/SPSB1/SOCS3/MAP3K8/FJX1/EFNA1/SGK1/VEGFA/ATF3/TRIB1/CD80/TNF/SIK1/CD83/LITAF/CXCL6/CD44/PLK2/REL/DUSP4/CCL20/DUSP2/LIF/RELB/ICOSLG/ICAM1/MXD1/GFPT2/PDLIM5/CLCF1
HALLMARK_UV_RESPONSE_DN	79	-0.438109717	-1.60108	0.009054151	tags=42%, list=20%, signal=34%	MRPS31/MAP1B/BDNF/SPOP/ANXA4/GRK5/NIPBL/ATRX/NOTCH2/IGFBP5/CDC42BPA/SMAD7/SRI/YTHDC1/DBP/PMP22/MET/PHF3/LTBP1/TJP1/SCAF8/ADD3/DUSP1/IRS1/NFIB/CDON/PTGFR/AMPH/FBLN5/HAS2/LPAR1/VLDLR/PLPP3
HALLMARK_COAGULATION	91	0.463003361	1.693168	0.009054151	tags=41%, list=14%, signal=35%	MMP1/APOC1/VWF/PLEK/COMP/DPP4/CTSB/FN1/HTRA1/CTSH/OLR1/TIMP1/CFI/A2M/ADAM9/MMP11/DUSP14/THBS1/C2/MMP14/PDGFB/PF4/DUSP6/WDR1/CTSK/LGMN/PECAM1/MMP8/BMP1/ITIH1/CTSV/CFB/F2/ARF4/CSRP1/MMP15/LRP1
HALLMARK_HYPOXIA	124	0.394077905	1.51147	0.019175571	tags=28%, list=9%, signal=26%	HMOX1/AMPD3/STC1/PLAC8/CXCR4/CA12/ERO1A/MIF/PIM1/ISG20/KDELR3/EFNA1/LOX/GPI/VEGFA/HAS1/ATF3/ADORA2B/PDGFB/IGFBP3/TPI1/GLRX/ETS1/TMEM45A/ADM/IGFBP1/LDHA/NAGK/GPC4/MT2A/PGF/HEXA/NOCT/TES/PGK1
HALLMARK_APOPTOSIS	102	0.415631482	1.53898	0.031453202	tags=22%, list=8%, signal=20%	HMOX1/CD2/CD14/KRT18/IL18/PRF1/CD69/LUM/GPX1/TIMP1/ISG20/BID/HGF/ATF3/BMF/TNF/CD44/IFNGR1/TOP2A/IGF2R/BAX/CD38
