# Supplementary material for: Potential biomarkers and immune cell infiltration involved in aortic valve calcification identified through integrated bioinformatics analysis
Source: Front Physiol. 2022 Dec 15;13:944551. doi: 10.3389/fphys.2022.944551 (PMC9797982; doi:10.3389/fphys.2022.944551)
Supplement: Supplementary file 2 [file DataSheet3.PDF]

Supplementary Material

1 Supplementary Figure 1

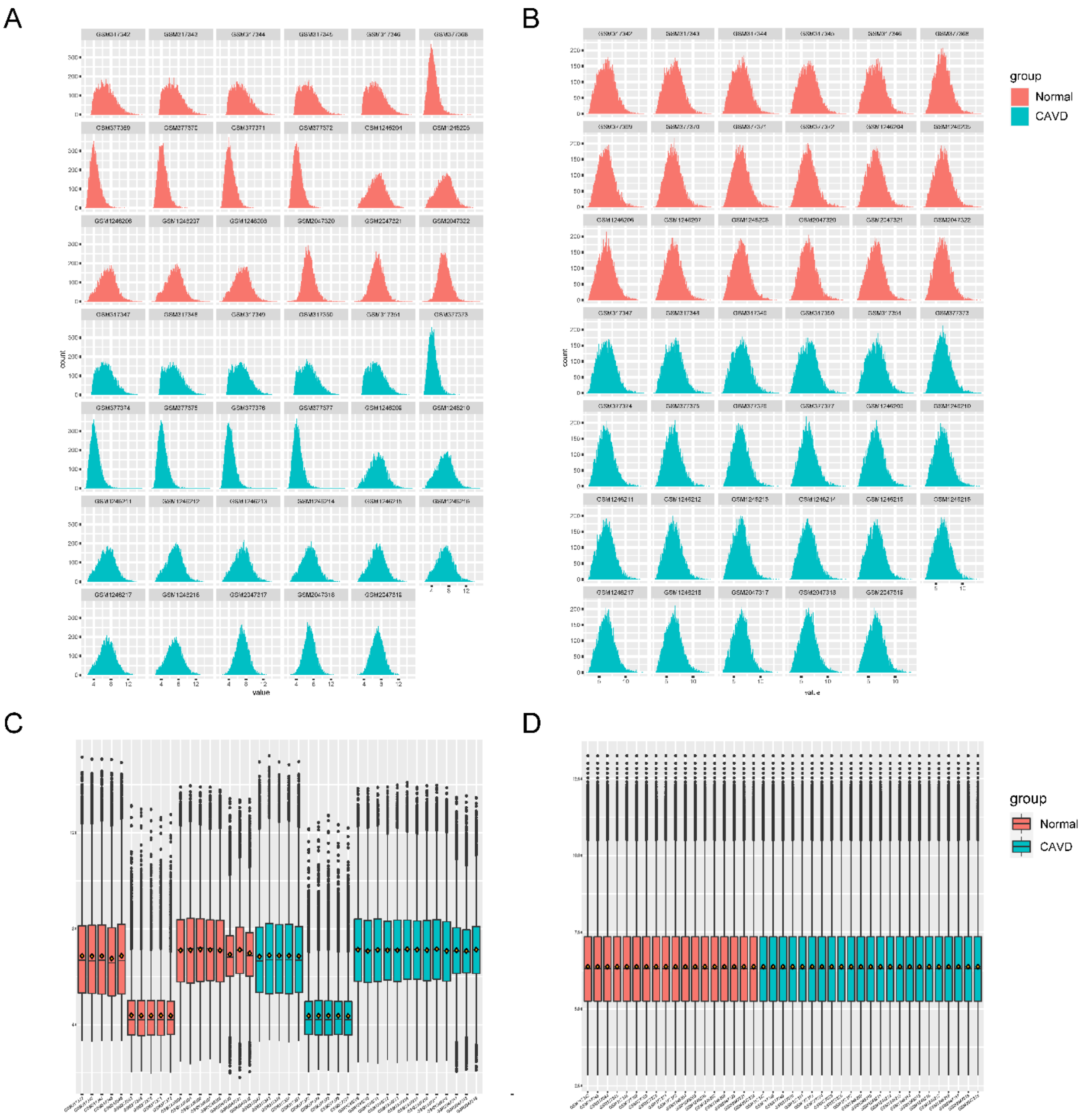

Supplementary Figure 1 | Comparison of data distribution before **(A, C)** and after **(B, D)** data correction.

2 Supplementary Figure 2

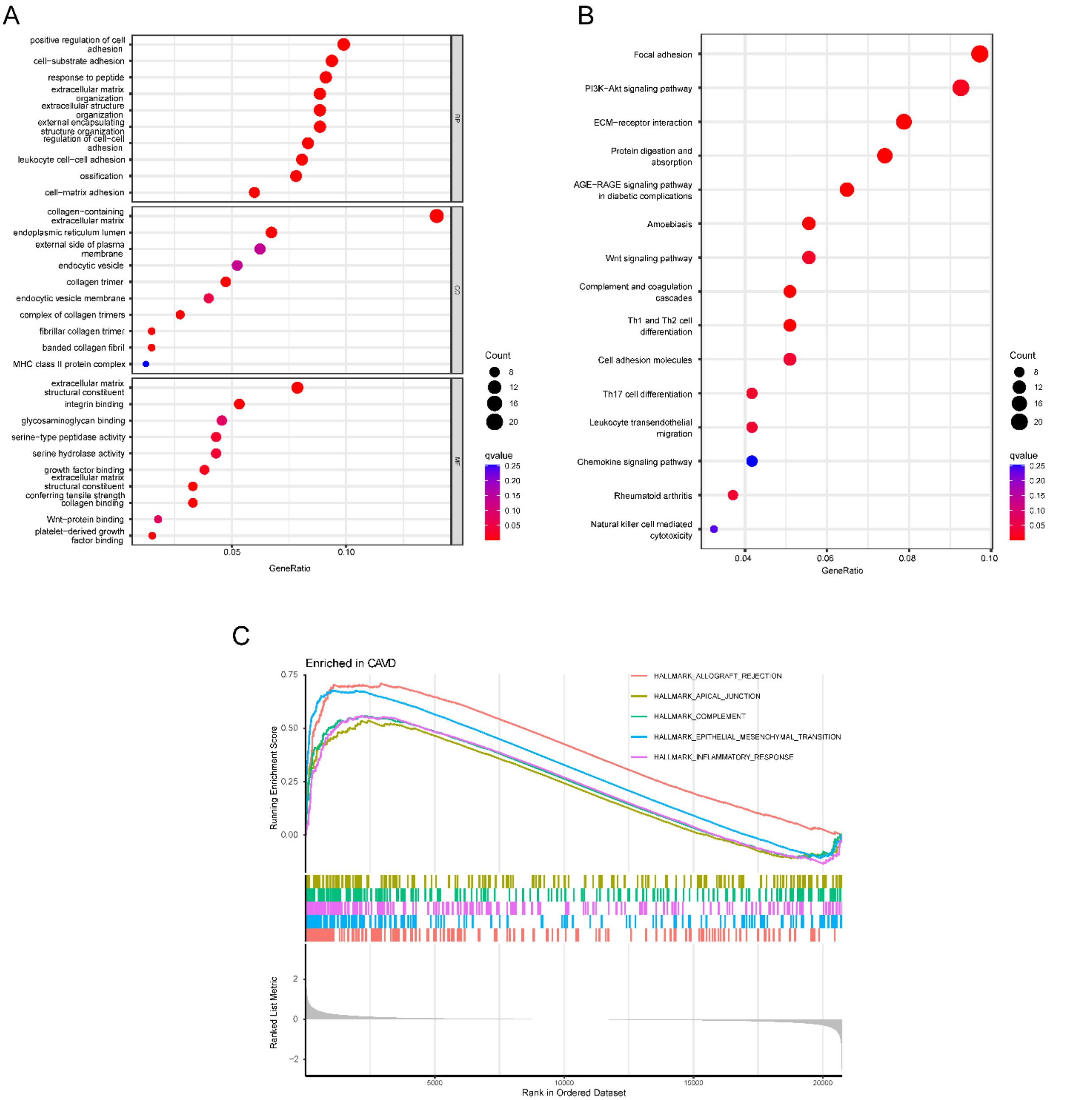

Supplementary Figure 2 | Biofunctional enrichment analysis of differentially expressed genes (DEGs) in validation dataset (GSE83453). (A) Gene Ontology functional enrichment analyses of DEGs and the top 10 biological process (BP), cellular component (CC), and molecular function (MF) terms. (B) Kyoto Encyclopedia of Genes and Genomes pathway enrichment analyses of DEGs. (C) Results of gene set enrichment analysis (GSEA) showing the top five hallmark pathways in CAVD gourp.

3 Supplementary Figure 3

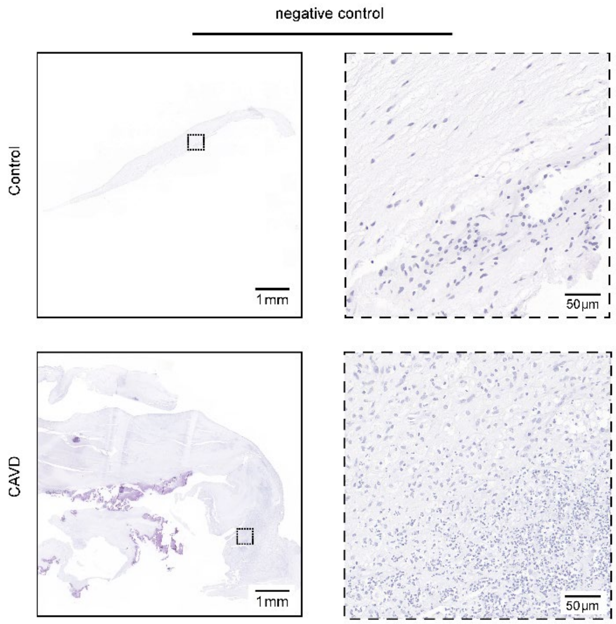

Supplementary Figure 3 | Immunohistochemical staining of human aortic valve tissue sections with omitted primary antibody as a negative control. The macro and micro staining results are shown from left to right.
