# Supplementary material for: Potential biomarkers and immune cell infiltration involved in aortic valve calcification identified through integrated bioinformatics analysis
Source: Front Physiol. 2022 Dec 15;13:944551. doi: 10.3389/fphys.2022.944551 (PMC9797982; doi:10.3389/fphys.2022.944551)
Supplement: Supplementary file 3 [file DataSheet1.PDF]

Table S1: The value of differential gene expression analysis results

symbol	logFC	AveExpr	t	P.Value	adj.P.Val
SCG2	1.600987	8.456166	7.240803	6.89E-09	4.31E-05
CD93	1.066685	8.853719	6.326193	1.39E-07	0.000293
SPP1	2.693645	8.697022	5.462969	2.39E-06	0.001302
S100A9	1.086501	7.62765	5.430331	2.66E-06	0.001333
TREM1	1.355312	7.6182	5.415018	2.80E-06	0.001348
CCL19	1.328693	9.049005	5.391547	3.03E-06	0.001402
PPBP	1.93579	5.468177	5.265803	4.56E-06	0.001587
MMP12	2.809928	6.768391	5.17742	6.09E-06	0.001949
ADRA2A	1.147034	6.488666	5.053934	9.09E-06	0.00242
WIF1	-1.394	6.577335	-4.99989	1.08E-05	0.002699
CHST9	-1.03588	5.453265	-4.80793	2.01E-05	0.004019
CXCL5	1.211717	4.628898	4.73559	2.54E-05	0.004543
S100A8	1.417874	6.120634	4.708694	2.77E-05	0.004691
CHI3L1	1.408215	7.072276	4.672487	3.10E-05	0.00501
IBSP	1.633427	6.515871	4.640088	3.44E-05	0.005382
ANGPTL7	-1.10435	7.297992	-4.54346	4.68E-05	0.006226
CTHRC1	1.167446	9.723622	4.507856	5.24E-05	0.006823
CD52	1.010367	6.803078	4.496887	5.42E-05	0.006987
C6	-1.18382	7.772961	-4.38005	7.83E-05	0.008468
TSPAN8	-1.2702	9.121841	-4.36585	8.19E-05	0.008728
NDNF	-1.06077	6.771636	-4.35872	8.37E-05	0.008728

**GPR83**	**-1.13416**	**6.941789**	**-4.17568**	**0.000148**	**0.01251**
**STMN2**	**1.278738**	**6.371065**	**4.091426**	**0.000192**	**0.01446**
**HMOX1**	**1.284154**	**8.908652**	**3.982822**	**0.000267**	**0.01789**
**TMEM132C**	**-1.04762**	**7.11874**	**-3.95696**	**0.000289**	**0.018325**
**MMP1**	**1.704455**	**5.156945**	**3.943054**	**0.000302**	**0.018325**
**AQP9**	**1.004729**	**6.097939**	**3.937966**	**0.000306**	**0.018431**
**VAT1L**	**-1.11022**	**8.654056**	**-3.87591**	**0.00037**	**0.019824**
**ANXA3**	**-1.04531**	**7.212297**	**-3.74174**	**0.000552**	**0.02472**
**TRHDE**	**-1.14889**	**6.162234**	**-3.67988**	**0.000663**	**0.027571**
**TM4SF18**	**1.068177**	**5.436166**	**3.64766**	**0.00073**	**0.028971**
**CHRDL1**	**-1.11525**	**7.716556**	**-3.57397**	**0.000905**	**0.032732**
**PRG4**	**1.161621**	**8.261438**	**3.406713**	**0.001468**	**0.043199**
**PDZRN4**	**-1.22251**	**7.195437**	**-3.32632**	**0.001845**	**0.049838**
